# An explainable machine learning model for type B aortic dissection identification: development and internal evaluation

**DOI:** 10.3389/fpubh.2026.1845404

**Published:** 2026-07-23

**Authors:** Donglin Li, Dilinuerkezi Abulimiti, Zaiying Yeerbao, Xiangxiang Ru, Xinxi Li, Lei Zhang, Jingdong Tang, Shuai Jiang, Ye Tian

**Affiliations:** 1 Department of Vascular Surgery, The First Affiliated Hospital of Xinjiang Medical University, Urumqi, China; 2 Department of Vascular Surgery, Shanghai Pudong Hospital, Fudan University, Shanghai, China

**Keywords:** internal evaluation, machine learning, neural network, shap, type B aortic dissection

## Abstract

**Purpose:**

Type B aortic dissection (TBAD) is a life-threatening cardiovascular emergency that requires timely recognition. This study aimed to develop and internally evaluate an explainable machine learning model for identifying existing TBAD using routinely available clinical history and admission laboratory data.

**Methods:**

This single-center retrospective case-control study included 1,640 participants, comprising 854 patients with CTA-confirmed TBAD and 786 hospitalized controls who underwent whole-aorta CTA and were confirmed not to have aortic dissection. Demographic characteristics, medical history, and admission laboratory test results were collected as 38 initial candidate features. Least absolute shrinkage and selection operator (LASSO) regression was applied to identify key discriminative variables. Machine-learning models based on eight algorithms were then developed and compared: support vector machine (SVM), gradient boosting machine (GBM), neural network, extreme gradient boosting (XGBoost), k-nearest neighbors (KNN), adaptive boosting (AdaBoost), light gradient boosting machine (LightGBM), and categorical boosting (CatBoost). Hyperparameters were tuned in the training set using grid search and repeated 10-fold cross-validation with five repeats. Model discrimination was evaluated in the held-out internal test set using the area under the receiver operating characteristic curve (AUC). The final representative model was selected for exploratory interpretation using SHapley Additive exPlanations (SHAP).

**Results:**

LASSO regression identified five predictors: hypertension, white blood cell count, lymphocyte percentage, basophil percentage, and monocyte count. Several models showed comparable discrimination in the held-out internal test set. The neural network model achieved an AUC of 0.883 in the training set and 0.852 in the held-out internal test set, with 95% confidence intervals of 0.864–0.902 and 0.819–0.885, respectively. Based on the ROC curve analysis, the neural network model showed relatively favorable discrimination and was therefore selected as the final representative model for exploratory SHAP-based interpretation. SHAP analysis indicated that lymphocyte percentage, hypertension, monocyte count, white blood cell count, and basophil percentage were the major contributors to the predictions of the final model, with lymphocyte percentage showing the highest mean absolute SHAP value.

**Conclusion:**

This study developed and internally evaluated an explainable machine-learning model for identifying existing TBAD in a single-center CTA-confirmed retrospective case-control cohort. Given the lack of external validation, this study should be regarded as exploratory. External validation in clinically relevant acute symptomatic populations is required before clinical implementation.

## 1 Introduction

Aortic dissection (AD) is a life-threatening cardiovascular emergency characterized by rapid mortality post-onset, underscoring the critical need for early and accurate diagnosis (1). Computed tomography (CT) is the preferred initial imaging modality when AD is suspected (2). As research progresses (3), various classification systems have emerged based on anatomical involvement and tear location (4). The Stanford classification, which categorizes AD into Type A (involving the ascending aorta) and Type B (confined to the descending aorta), is the most widely used (5). The global clinical detection rate and disease burden of AD have risen significantly in recent years.

Acute aortic dissection is characterized by sudden onset and high mortality. Type B aortic dissection (TBAD) represents a particular diagnostic challenge because its early clinical manifestations are often heterogeneous and atypical (6). In some patients, TBAD may mimic other acute cardiovascular, pulmonary, or abdominal conditions, such as acute coronary syndrome or pulmonary embolism, which may contribute to diagnostic delays and missed opportunities for timely treatment. Therefore, it may be useful to explore whether routinely available clinical and laboratory data contain discriminative information related to existing TBAD before confirmatory imaging.

At present, the diagnosis of TBAD mainly relies on computed tomography angiography (CTA), which is widely used as the primary confirmatory imaging modality (9). In clinical practice, CTA may not always be performed immediately because of patient transfer, scanner availability, preparation procedures, or workflow-related delays. Therefore, this study explored whether routinely available clinical information and admission laboratory data could be used to develop an explainable machine-learning model for identifying existing TBAD before confirmatory imaging. Given the retrospective case-control design and lack of external validation, this model should be regarded as exploratory rather than a clinically deployable tool.

Machine learning has received increasing attention in clinical research because of its ability to model complex and non-linear relationships in routinely collected data (7). Previous studies have explored machine-learning approaches for the diagnosis or risk assessment of acute critical illnesses (8), including aortic dissection (10–12). Some studies have also incorporated explainable methods, such as SHapley Additive exPlanations (SHAP), to interpret model predictions in the context of aortic dissection. However, evidence remains limited regarding explainable machine-learning models focused specifically on existing TBAD using routinely available clinical history and admission laboratory variables. Therefore, this single-center retrospective case-control study was conducted as an exploratory analysis to develop and internally evaluate an explainable machine-learning model for identifying existing TBAD. Multiple machine-learning algorithms were compared, and SHAP analysis was used to describe the relative contribution of individual predictors to model output.

## 2 Materials and methods

### 2.1 Study design and data source

**Figure 1:**
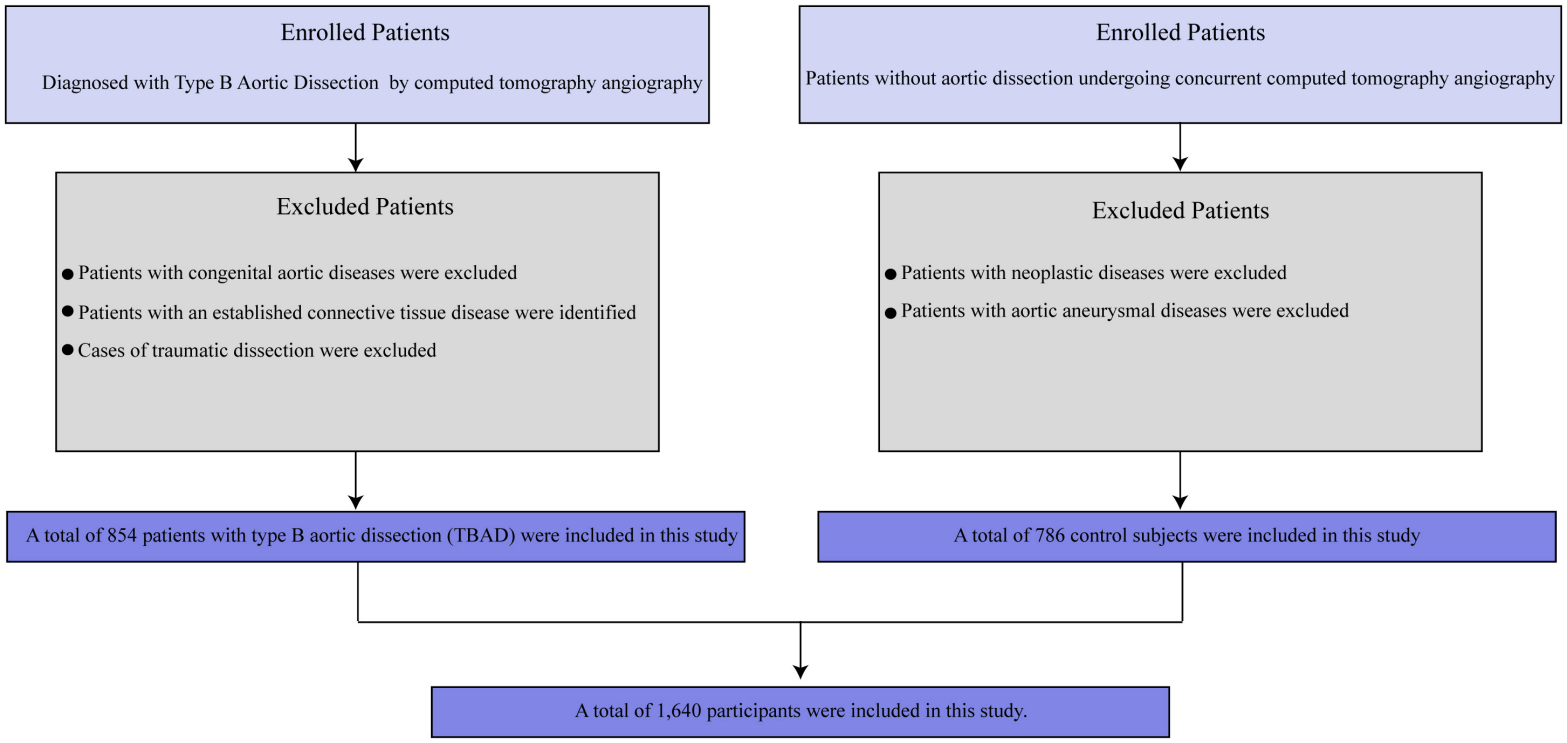
Flowchart illustrating the inclusion and exclusion criteria for study participants.

This single-center retrospective case-control study analyzed data from patients treated at the First Affiliated Hospital of Xinjiang Medical University between 2012 and 2025. The case group included 854 patients with type B aortic dissection (TBAD) confirmed by computed tomography angiography (CTA). The control group included 786 hospitalized patients who underwent whole-aorta CTA during the same study period and were confirmed to have no evidence of aortic dissection. Demographic characteristics, medical history, and laboratory test results were extracted from the electronic medical records. A total of 38 candidate variables were initially evaluated. The inclusion and exclusion criteria are shown in Figure 1.

### 2.2 Inclusion criteria

Patients were included in the TBAD group if they had a diagnosis of TBAD confirmed by CTA during hospitalization. Control subjects were included if they were hospitalized patients who underwent whole-aorta CTA during the same study period and had no imaging evidence of aortic dissection.

### 2.3 Exclusion criteria

Patients with congenital aortic diseases, diagnosed connective tissue diseases, or traumatic aortic dissection were excluded.

### 2.4 Data splitting, missing data, and feature selection

After applying the inclusion and exclusion criteria, the dataset was randomly divided into a training set and a held-out internal test set at a ratio of 7:3 using stratified sampling according to TBAD status. Missing data were handled after the dataset was divided into the training and held-out internal test sets to reduce the risk of information leakage. Chained-equation imputation was performed using the mice package in R, version 3.18.0, with predictive mean matching as the imputation method and a maximum of 10 iterations. Feature selection was then performed in the training set using least absolute shrinkage and selection operator regression with 10-fold cross-validation. Variables with non-zero coefficients at lambda.1se were retained for model development.

### 2.5 Model development and internal evaluation

Machine-learning models based on eight algorithms were developed and compared: support vector machine (SVM), gradient boosting machine (GBM), neural network, extreme gradient boosting (XGBoost), k-nearest neighbors (KNN), adaptive boosting (AdaBoost), light gradient boosting machine (LightGBM), and categorical boosting (CatBoost). Hyperparameters were tuned within the training set using grid search and repeated 10-fold cross-validation with five repeats. Model discrimination was assessed in the held-out internal test set using the area under the receiver operating characteristic curve (AUC).

### 2.6 Model performance assessment

Model performance was evaluated using the area under the receiver operating characteristic curve (AUC), accuracy, sensitivity, specificity, precision, and F1-score. Calibration curves and decision curve analysis were generated as exploratory supplementary assessments.

### 2.7 Model interpretation

The SHapley Additive exPlanations (SHAP) framework was used for exploratory interpretation of the final representative model. Feature importance rankings and SHAP summary plots were generated to describe the relative contribution and direction of influence of individual predictors on model output.

### 2.8 Statistical analysis

Categorical variables are presented as numbers and percentages. Continuous variables with skewed distributions are summarized as medians with interquartile ranges. Between-group comparisons of baseline characteristics were performed using the chi-square test for categorical variables and the Wilcoxon rank-sum test for continuous variables. All statistical analyses and machine learning modeling were conducted in R, version 4.5.1. The caret package, version 7.0.1, was used for model development. A two-sided *P* value < 0.05 was considered statistically significant.

## 3 Results

### 3.1 Characteristics of the study population

A total of 1,640 participants were included in this study, comprising 854 patients with CTA-confirmed TBAD and 786 control subjects who underwent whole-aorta CTA and showed no imaging evidence of aortic dissection. After applying the inclusion and exclusion criteria, the dataset was randomly divided into a training set and a held-out internal test set at a ratio of 7:3. The overall study population included 1,277 males and 363 females. Detailed baseline characteristics of the TBAD and control groups are presented in Table 1.

**Table 1:** Baseline characteristics.

Characteristic	No	Yes	*p*.overall
	*N* = 786	*N* = 854	
Gender			0.003
Male	587 (74.68%)	690 (80.80%)	
Female	199 (25.32%)	164 (19.20%)	
Age	59.00 [51.00;66.00]	56.00 [50.00;64.00]	0.002
Hypertension			< 0.001
No hypertension	448 (57.00%)	181 (21.19%)	
Has high blood pressure	338 (43.00%)	673 (78.81%)	
Smoking, history			0.001
Does not smoke	538 (68.45%)	519 (60.77%)	
Smoking	248 (31.55%)	335 (39.23%)	
Diabetes, mellitus			< 0.001
No diabetes	684 (87.02%)	794 (92.97%)	
Have diabetes	102 (12.98%)	60 (7.03%)	
White, blood, cell, count	6.40 [5.21; 7.84]	9.39 [7.18; 12.27]	< 0.001
Neutrophil, percentage	59.15 [52.60; 66.60]	75.45 [65.23; 82.70]	< 0.001
Lymphocyte, percentage	29.40 [22.60; 36.00]	14.00 [8.80; 23.40]	< 0.001
Monocyte, percentage	7.60 [6.30; 9.30]	8.00 [6.30; 9.90]	0.009
Eosinophil, percentage	1.90 [1.20; 3.10]	0.70 [0.10; 1.90]	< 0.001
Basophil, percentage	0.50 [0.30; 0.70]	0.30 [0.20; 0.40]	< 0.001
Neutrophil, count	3.71 [2.84; 4.86]	6.96 [4.76; 9.77]	< 0.001
Lymphocyte, count	1.81 [1.40; 2.25]	1.36 [0.94; 1.86]	< 0.001
Monocyte, count	0.48 [0.37; 0.61]	0.73 [0.52; 1.01]	< 0.001
Eosinophil, count	0.12 [0.07; 0.20]	0.06 [0.01; 0.15]	< 0.001
Basophil, count	0.03 [0.02; 0.04]	0.03 [0.02; 0.04]	< 0.001
Red, blood, cell, count	4.65 [4.23; 5.03]	4.49 [4.06; 4.85]	< 0.001
Hemoglobin	141.00 [127.00; 150.00]	135.00 [123.00; 147.00]	< 0.001
Mean, corpuscular, volume	91.00 [87.90; 94.10]	90.70 [87.80; 93.60]	0.173
Mean, corpuscular, hemoglobin	30.20 [29.00; 31.30]	30.20 [29.10; 31.30]	0.895
Red, blood, cell, distribution, width	13.00 [12.40; 13.70]	13.00 [12.40; 13.60]	0.246
Mean, platelet, volume	10.40 [9.80; 11.10]	10.10 [9.60; 10.90]	< 0.001
Plateletcrit	0.22 [0.18; 0.26]	0.21 [0.17; 0.25]	< 0.001
Platelet, distribution, width	12.40 [10.90; 15.20]	11.60 [10.30; 14.00]	< 0.001
Platelet, count	207.50 [173.00; 254.00]	201.00 [156.00; 251.00]	0.03
Serum, potassium	3.76 [3.55; 4.01]	3.65 [3.35; 3.95]	< 0.001
Serum, chloride	105.70 [103.30; 107.90]	104.20 [101.60; 106.57]	< 0.001
Serum, bicarbonate	25.59 [23.56; 27.61]	25.00 [22.80; 27.14]	< 0.001
Serum, magnesium	0.87 [0.81; 0.92]	0.88 [0.81; 0.94]	0.052
Serum, phosphorus	1.19 [1.06; 1.32]	1.09 [0.92; 1.25]	< 0.001
Urea	5.78 [4.80; 7.08]	5.93 [4.80; 7.53]	0.083
Blood, glucose	4.98 [4.42; 6.15]	6.22 [5.20; 7.34]	< 0.001
High, density, lipoprotein	0.94 [0.77; 1.17]	0.88 [0.72; 1.10]	< 0.001
Total, bilirubin	13.27 [10.05; 17.90]	16.31 [11.83; 23.08]	< 0.001
Direct, bilirubin	1.87 [0.30; 3.90]	0.49 [0.30; 3.99]	0.146
Indirect, bilirubin	9.12 [6.18; 12.98]	11.24 [7.13; 17.18]	< 0.001
Albumin	39.90 [37.21; 42.97]	38.30 [34.70; 41.58]	< 0.001
Osmolality	286.90 [274.15; 294.18]	280.62 [271.55; 288.81]	< 0.001

### 3.2 Feature selection

**Figure 2:**
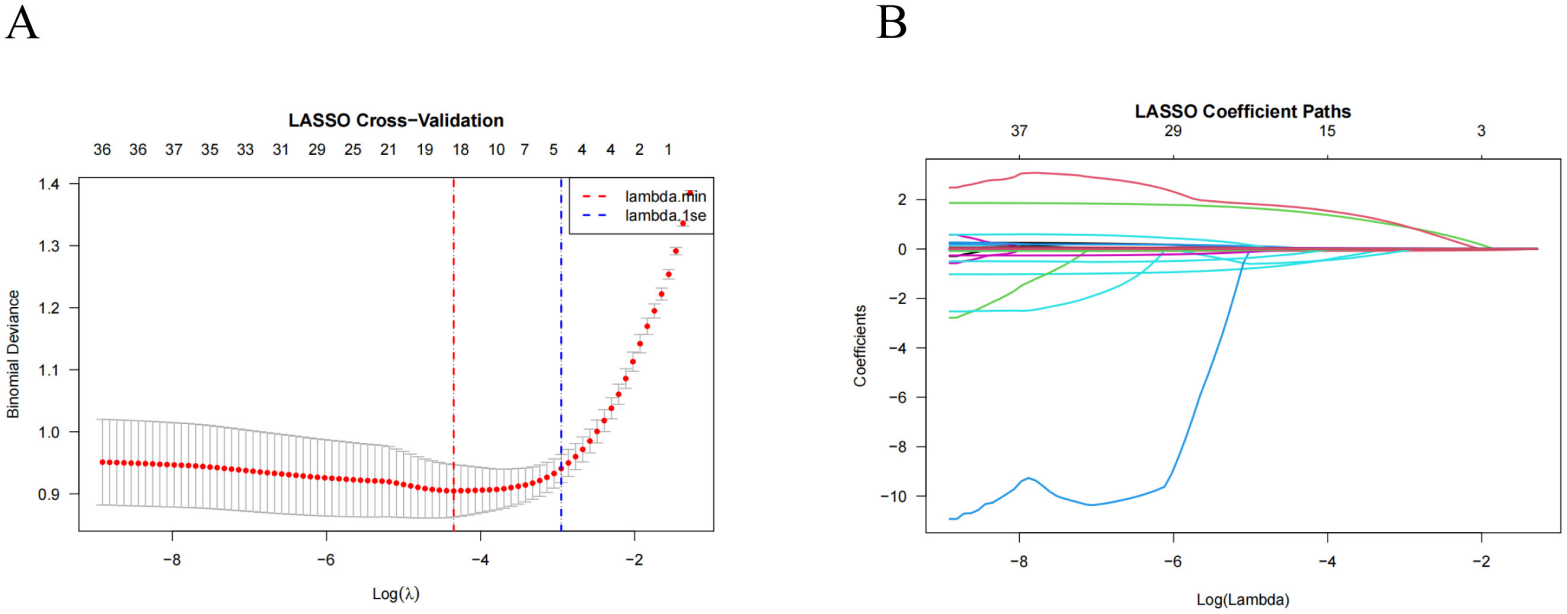
LASSO regression-based variable screening. (**A**) Ten-fold cross-validation plot for selecting the optimal penalty parameter, lambda, in the LASSO model. (**B**) LASSO coefficient profiles of candidate variables.

Feature selection was performed in the training set using least absolute shrinkage and selection operator (LASSO) regression with 10-fold cross-validation. Variables with non-zero coefficients at lambda.1se were retained for subsequent model development, as illustrated in Figure 2. Five predictors were selected: history of hypertension, white blood cell count, lymphocyte percentage, basophil percentage, and monocyte count. These variables were used as input features for the machine-learning models.

### 3.3 Model performance evaluation

**Figure 3:**
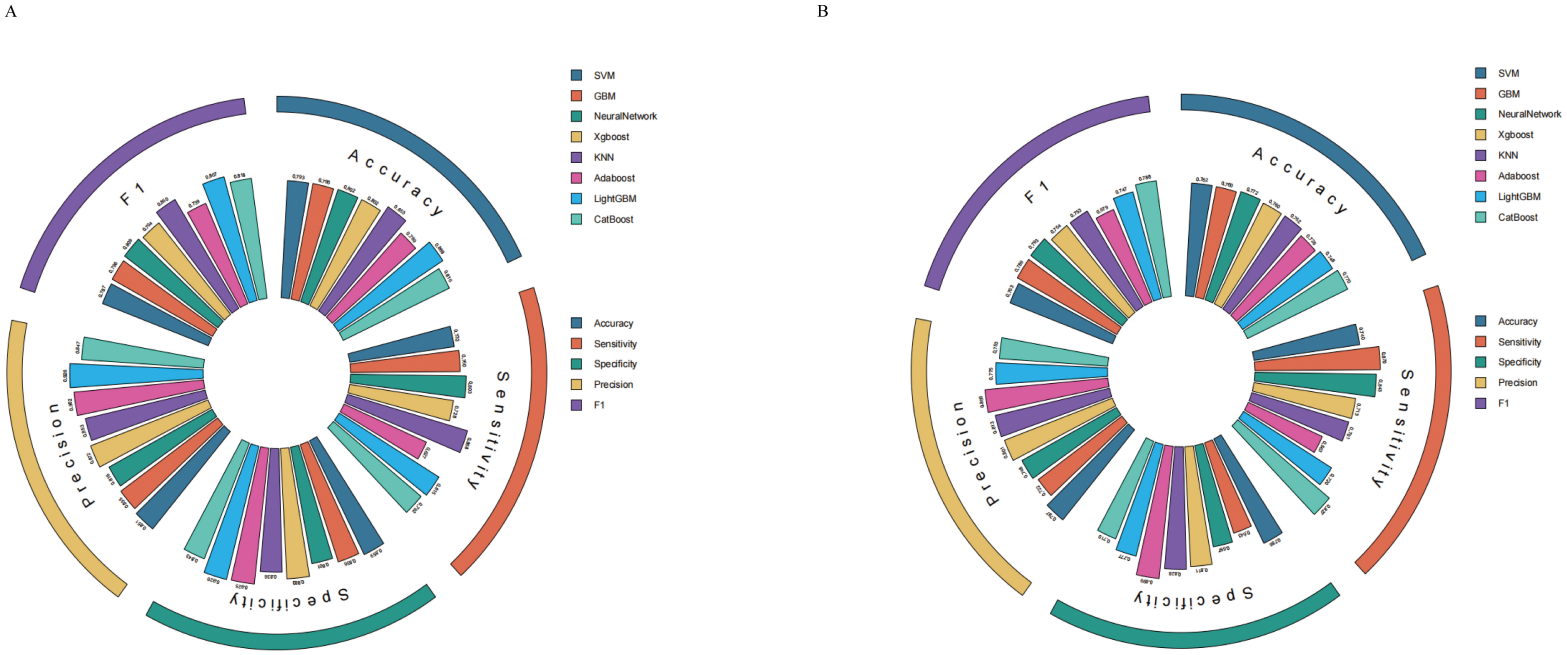
Classification performance metrics of the machine-learning models. (**A**) Performance in the training set. (**B**) Performance in the held-out internal test set.

**Figure 4:**
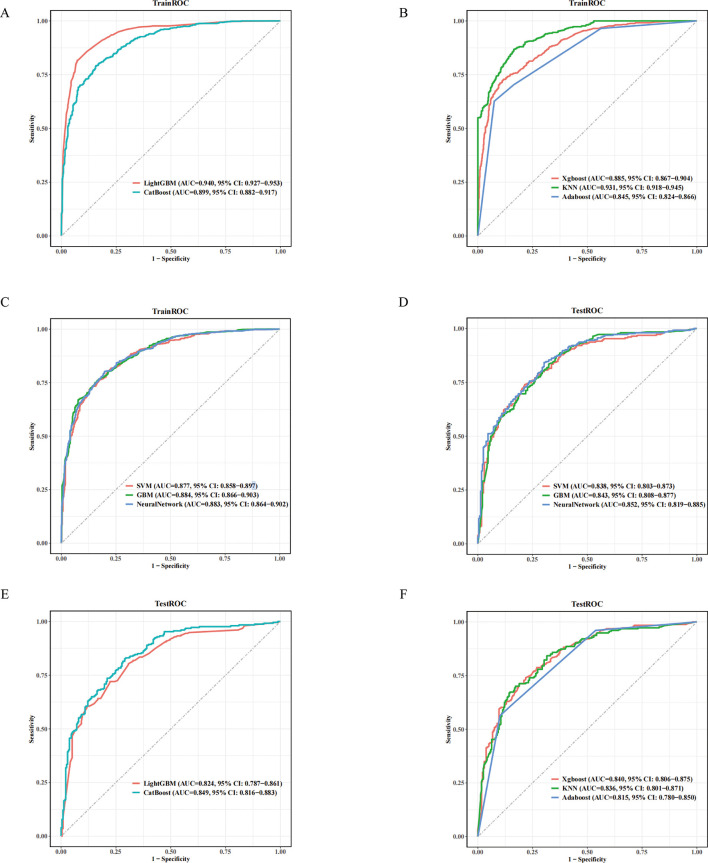
Receiver operating characteristic curves of the machine-learning models in the training set and held-out internal test set. (**A–C**) ROC curves in the training set. (**D–F**) ROC curves in the held-out internal test set. AUC values with 95% confidence intervals are shown in the legends.

**Figure 5:**
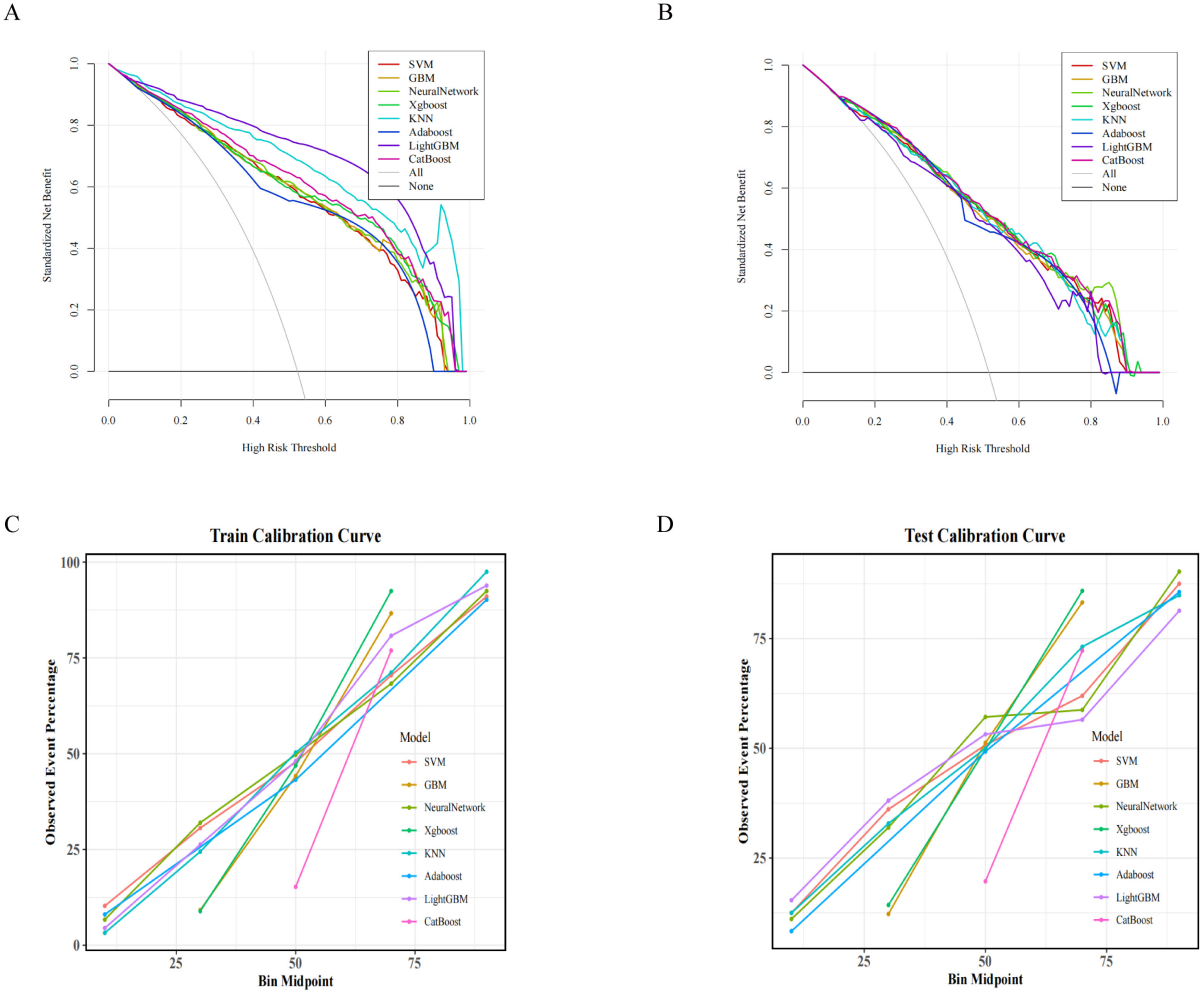
Decision curve analysis and calibration curves of the machine-learning models in the training set and held-out internal test set. (**A**) Decision curve analysis in the training set. (**B**) Decision curve analysis in the held-out internal test set. (**C**) Calibration curves in the training set. (**D**) Calibration curves in the held-out internal test set.

Machine-learning models based on eight algorithms were developed to identify TBAD and were evaluated in both the training set and the held-out internal test set. Threshold-dependent performance metrics, including accuracy, sensitivity, specificity, precision, and F1-score, are summarized in Table 2, 3 and visualized in Figure 3. Receiver operating characteristic curves and AUC values are shown in Figure 4. Exploratory decision curve analysis and calibration curves are shown in Figure 5.

**Table 2:** Performance of candidate models in the training set.

Model	Threshold	Accuracy	Sensitivity	Specificity	Precision	F1
SVM	0.598	0.793	0.732	0.859	0.851	0.787
GBM	0.521	0.796	0.76	0.836	0.835	0.796
Neural network	0.503	0.802	0.803	0.801	0.816	0.809
Xgboost	0.539	0.802	0.728	0.883	0.872	0.794
KNN	0.473	0.853	0.868	0.836	0.853	0.86
Adaboost	0.77	0.769	0.627	0.925	0.902	0.739
LightGBM	0.545	0.869	0.815	0.929	0.926	0.867
Catboost	0.624	0.816	0.792	0.843	0.847	0.818

**Table 3:** Performance of candidate models in the held-out internal test set.

Model	Threshold	Accuracy	Sensitivity	Specificity	Precision	F1
SVM	0.537	0.762	0.74	0.786	0.787	0.763
GBM	0.427	0.76	0.87	0.643	0.722	0.789
Neural network	0.425	0.772	0.843	0.697	0.748	0.793
Xgboost	0.526	0.76	0.713	0.811	0.801	0.754
KNN	0.601	0.762	0.701	0.828	0.813	0.753
Adaboost	0.77	0.726	0.563	0.899	0.856	0.679
LightGBM	0.691	0.748	0.72	0.777	0.775	0.747
Catboost	0.608	0.77	0.827	0.71	0.753	0.788

In the held-out internal test set, several models showed comparable discrimination. The neural network model achieved an AUC of 0.883 in the training set and 0.852 in the held-out internal test set, with 95% confidence intervals of 0.864–0.902 and 0.819–0.885, respectively. For threshold-dependent metrics in the internal test set, the neural network model achieved an accuracy of 0.772, sensitivity of 0.843, specificity of 0.697, precision of 0.748, and F1-score of 0.793.

Compared with their training-set performance, some models showed a larger decrease in the held-out internal test set. For example, LightGBM achieved an accuracy of 0.869 and an F1-score of 0.867 in the training set, but these values decreased to 0.748 and 0.747, respectively, in the internal test set. KNN also decreased from a training accuracy of 0.853 and F1-score of 0.860 to a test accuracy of 0.762 and F1-score of 0.753. In contrast, the neural network model showed a smaller relative decrease between the training and internal test sets. Therefore, based on ROC curve analysis and internal model performance, it was selected as the final representative model for exploratory SHAP-based interpretation.

### 3.4 Interpretability of the selected final model

**Figure 6:**
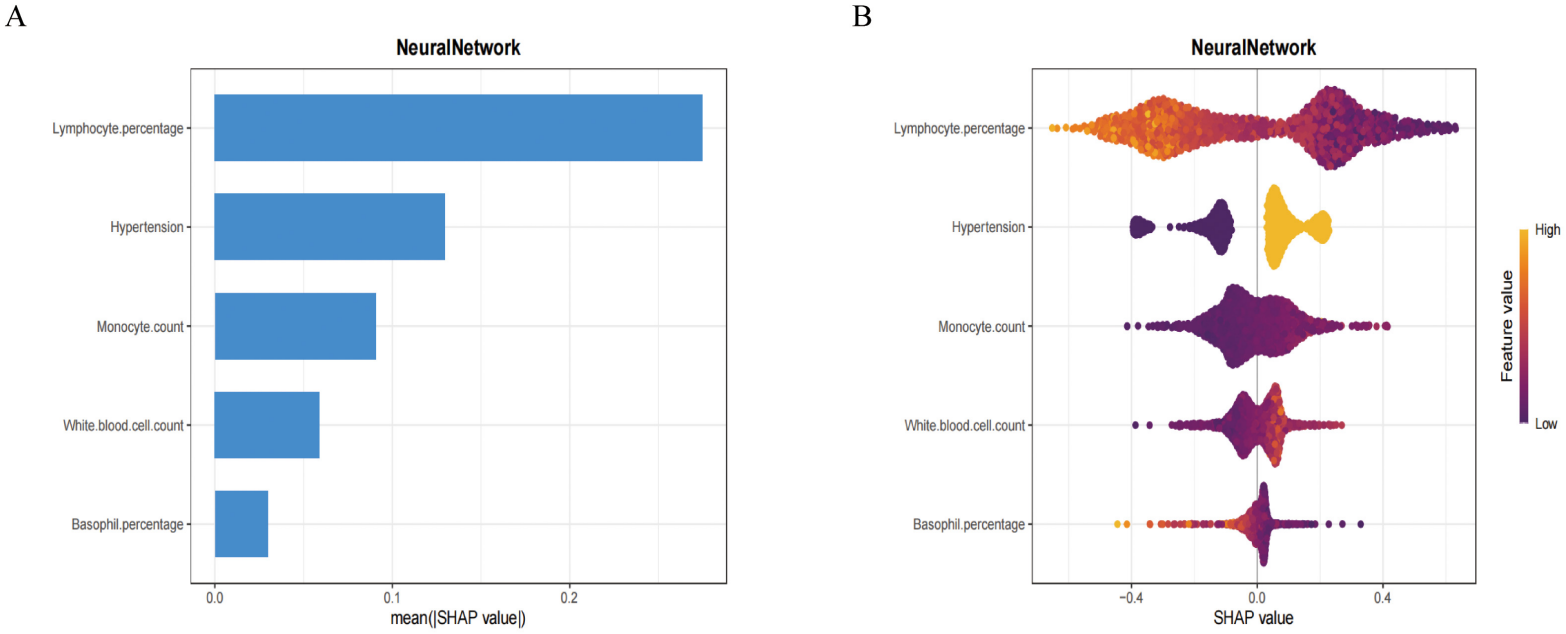
SHAP explanation plots for the neural network model. (**A**) SHAP bar plot. (**B**) SHAP beeswarm plot.

SHapley Additive exPlanations (SHAP) analysis was used for exploratory interpretation of the selected neural network model. SHAP values were used to describe the relative contribution of each predictor to the model output. As shown in Figure 6A, lymphocyte percentage had the highest mean absolute SHAP value, followed by history of hypertension, monocyte count, white blood cell count, and basophil percentage. These variables were identified as the main contributors to the predictions of the final representative model. The SHAP beeswarm plot in Figure 6B illustrates the distribution and direction of feature contributions at the population level.

**Figure 7:**
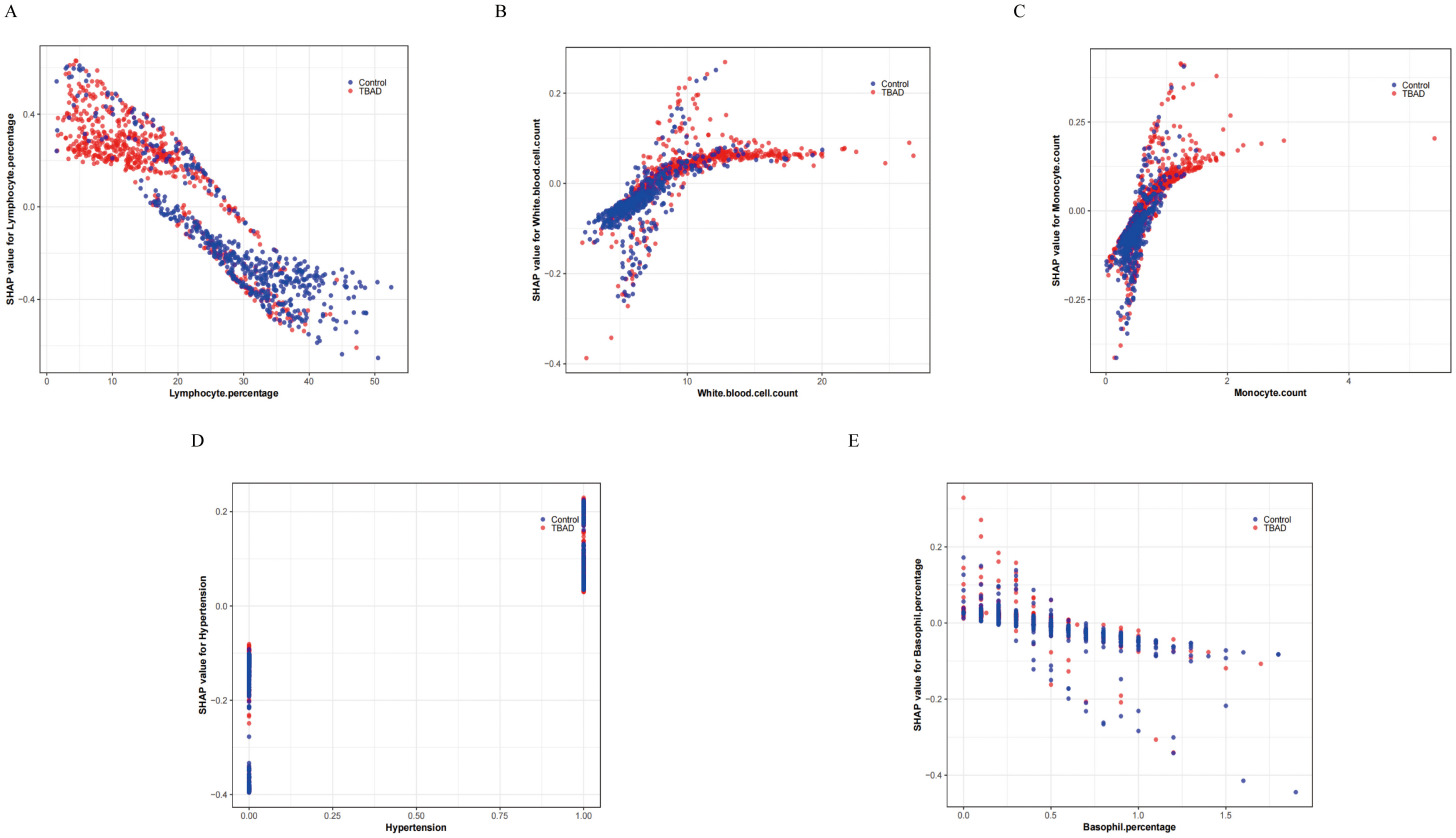
SHAP dependence plots showing the relationship between selected predictor values and their SHAP values in the final representative model. Points are colored according to the observed group, allowing visualization of feature distributions in TBAD and control subjects. (**A**) Lymphocyte percentage. (**B**) White blood cell count. (**C)** Monocyte count. (**D**) Hypertension. (**E**) Basophil percentage.

In general, lower lymphocyte percentage, the presence of hypertension, and higher monocyte count were associated with a higher predicted probability of TBAD. The relationships between actual feature values and SHAP values for the selected predictors are shown in Figure 7.

## 4 Discussion

In this study, we developed and internally evaluated an exploratory machine-learning model for identifying existing TBAD using basic medical history and routine blood test results. Eight machine-learning algorithms were compared. Based on ROC curve analysis, the neural network model showed relatively favorable discrimination, achieving an AUC of 0.852 in the held-out internal test set.

In this study, lymphocyte percentage, monocyte count, and hypertension were identified as important variables associated with model prediction. The onset of aortic dissection is typically triggered by a tear in the inner layer of the aorta (13). Dan Yu et al. (14) in a multicenter study, concluded that decreased lymphocyte levels are associated with the risk of death in acute aortic dissection. Their research suggests that, as indicated in previous studies, immune and inflammatory mechanisms are involved in the onset and progression of cardiovascular diseases (15, 16). Currently, immune dysregulation may be present in patients with acute aortic dissection (17). Other studies have also revealed the importance of immune cells (18–21), often manifested as abnormal expression of immune molecules. Immune cell infiltration has also been found in existing aortic specimens (22). In the present study, lower lymphocyte percentage was associated with a higher predicted probability of TBAD in the model. This finding suggests that lymphocyte percentage may contain discriminative information related to existing TBAD. In acute cardiovascular diseases, most studies attribute this association to systemic inflammatory responses (23–25); however, there are fewer reports directly linking lymphocyte percentage to disease occurrence. This study further supplements the potential role of lymphocyte percentage in cardiovascular disease-related modeling and may aid exploratory identification of existing TBAD. Furthermore, as it is a routine test item for emergency admission patients (26, 27), the required data can be easily obtained, making it suitable for retrospective investigation.

Monocyte count, as an inflammatory biomarker, may also reflect disease-related inflammatory activity in TBAD. When dissection occurs, platelets may be further activated (28), aggregating and releasing inflammatory factors into the blood vessels, which in turn activate monocytes (29). This process may contribute to the inflammatory response and disease progression. Yiping Chen et al. (30) in a single-center study, found that the combination of the lymphocyte-to-monocyte ratio, platelet count, and lymphocyte-to-neutrophil ratio could predict in-hospital mortality risk in patients with acute type A aortic dissection. In the present study, a higher monocyte count was observed among TBAD patients, which is consistent with its role as an inflammatory marker. However, whether it can meaningfully contribute to TBAD identification remains to be investigated in prospective studies.

Hypertension history, as a classical risk factor for aortic dissection (31), was also selected as an important variable in this study. This finding is consistent with previous clinical knowledge and supports the inclusion of routine medical history variables in the exploratory model.

Regarding model selection, the neural network model was retained for further analysis because it showed relatively favorable discrimination based on ROC curve analysis and acceptable performance in the held-out internal test set. Therefore, it was selected as the final representative model for exploratory SHAP-based interpretation.

This study has several limitations. As a retrospective single-center investigation, the findings are inherently preliminary. In addition, the model was only internally evaluated and lacked external validation. Therefore, the present study should be regarded as exploratory, and further external validation is required before clinical implementation.

## 5 Conclusion

This study developed and internally evaluated an explainable machine-learning model for identifying existing TBAD using routinely available clinical history and admission laboratory data. Given the retrospective design and lack of external validation, this study should be regarded as exploratory. External validation is required before clinical implementation.

## Data Availability

The datasets presented in this article are not readily available because the dataset used and analyzed in this study can be provided by the corresponding author upon reasonable request. Requests to access the datasets should be directed to Ye Tian (chinese1018@126.com).
